# *tert*-Butyl 6-benzoyl-5-hydr­oxy-2-oxo-2*H*-chromene-4-carboxyl­ate

**DOI:** 10.1107/S1600536808016188

**Published:** 2008-06-07

**Authors:** Robabeh Baharfar, S. Mohammad Vahdat, S. Meysam Baghbanian

**Affiliations:** a Department of Chemistry, University of Mazandaran, 47415, Babolsar, Iran

## Abstract

In the title compound, C_21_H_18_O_6_, a previously unknown coumarin derivative, the benzoyl substitutent makes a dihedral angle of 53.80 (16)° with the plane of the coumarin rings. An intramolecular O—H⋯O hydrogen bond is observed.

## Related literature

For related literature, see: Jurd *et al.* (1971 ▶); Kasinadhuni *et al.* (1999 ▶); Sardari *et al.* (1999 ▶); Soine (1964 ▶).
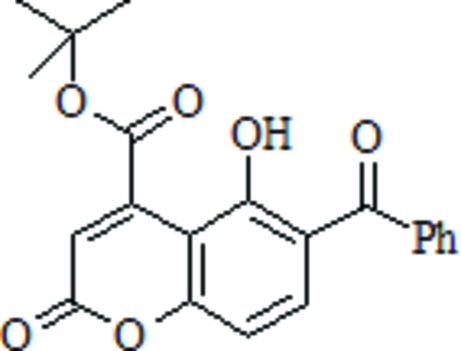


## Experimental

### 

#### Crystal data

C_21_H_18_O_6_
*M* *_r_* = 366.35
Monoclinic, 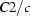

*a* = 22.1263 (12) Å
*b* = 7.3012 (4) Å
*c* = 22.5428 (12) Å
β = 103.118 (5)°
*V* = 3546.7 (3) Å^3^
*Z* = 8
Mo *K*α radiation
μ = 0.10 mm^−1^
*T* = 120 (2) K
0.25 × 0.20 × 0.20 mm

#### Data collection

Bruker SMART APEXII CCD area-detector diffractometer
Absorption correction: multi-scan (*APEX2*; Bruker, 2005 ▶) *T*_min_ = 0.972, *T*_max_ = 0.981
18827 measured reflections
4686 independent reflections
3022 reflections with *I* > 2σ(*I*)
*R*_int_ = 0.034

#### Refinement

*R*[*F* ^2^ > 2σ(*F* ^2^)] = 0.051
*wR*(*F* ^2^) = 0.132
*S* = 1.00
4686 reflections
247 parameters
H-atom parameters constrained
Δρ_max_ = 0.39 e Å^−3^
Δρ_min_ = −0.24 e Å^−3^

### 

Data collection: *APEX2* (Bruker, 2005 ▶); cell refinement: *APEX2*; data reduction: *APEX2*; program(s) used to solve structure: *SHELXTL* (Sheldrick, 2008 ▶); program(s) used to refine structure: *SHELXTL*; molecular graphics: *SHELXTL*; software used to prepare material for publication: *SHELXTL*.

## Supplementary Material

Crystal structure: contains datablocks I, global. DOI: 10.1107/S1600536808016188/at2560sup1.cif

Structure factors: contains datablocks I. DOI: 10.1107/S1600536808016188/at2560Isup2.hkl

Additional supplementary materials:  crystallographic information; 3D view; checkCIF report

## Figures and Tables

**Table 1:** Hydrogen-bond geometry (Å, °)

*D*—H⋯*A*	*D*—H	H⋯*A*	*D*⋯*A*	*D*—H⋯*A*
O3—H3*O*⋯O6	0.94	1.72	2.5365 (15)	143

**Fig. 1.**
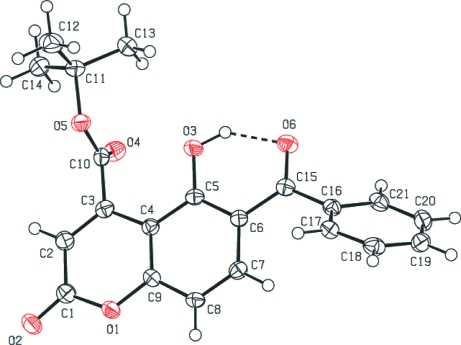
View of the title molecule showing the atomic numbering. Displacement ellipsoids are drawn at the 50° probability level for non-h atoms are shown as spheres of arbitrary radii.

## supplementary crystallographic information

### Comment

The coumarin nucleus is incorporated in many biologically active compounds and natural products. Coumarin and its derivatives have been found to show a wide range of bioactivities such as anticoagulant, estrogenic, molluscacidal, hypothermic (Soine, 1964), antimicrobial (Jurd *et al.*, 1971) anti-inflammatory, antifungal (Sardari *et al.*, 1999) and antinuclear activities (Kasinadhuni *et al.*, 1999). We have recently synthesized a series of 5-hydroxy and 7-hydroxy coumarins based on a direct, efficient and operationally convenient approach and we report here the synthesis and crystal structure of the title compound (I), which is one of the products of this reaction.

The molecular structure of (I) is illustrated in Fig 1. The inclinations of the planes of the t-butoxycarbonyl, hydroxy and benzoyl substitutents with respect to the coumarin ring system are 89.93, 3.15 and 53.80°, respectively. Dihedral angle between tert-butoxycarbonyl group and coumarin moiety is 89.97 (16)°. This deviation from the coumarin plane may be due to the steric repulsion between this bulky group and hydroxy group. Dihedral angle between hydroxy group and carbonyl of benzoyl group is -2.6 (2)°. Therefore, these groups are nearly coplanar and form an intramolecular O—H···O═C hydrogen bonding (Table 1). Dihedral angle between phenyl and carbonyl in benzoyl group is 132.65 (15)°.

### Experimental

To a magnetically stirred solution of 2,4-dihydroxy benzophenone (0.43 g, 2 mmol) and triphenylphosphine (0.52 g, 2 mmol) in 10 ml CH_2_Cl_2_ was added dropwise at 263 K over 10 min ditert-butyl acetylenedicarboxylate (0.45 g, 2 mmol). The reaction mixture was then allowed to warm up to room temperature and stand for 48 h. The solvent was removed under reduced pressure and the residue was separated by silica gel column chromatography (Merck 230–400 mesh) using n-hexane–ethyl acetate as eluent. Yellow Oil, yield 75\%. ^1^H NMR (500 MHz, CDCl_3_): δ = 1.62 (9 H, s, CMe_3_), 6.27 (1 H, s, CH), 6.83 (1 H, d, 3JHH = 8.9 Hz, CH), 7.51-7.70 (5 H, m, CH, aromatic), 7.79 (1 H, d, 3JHH = 8.9 Hz, CH), 13.72 (1 H, s, OH). ^13^C NMR (125.7 MHz, CDCl_3_): δ = 28.00 (CMe_3_), 84.50 (CMe_3_), 106.42 (CH), 108.18 (C), 112.87, 114.84 (2 CH), 128.67, 129.06 and 132.49 (3 CH), 136.92, 137.09 and 146.10 (3 C), 159.11, 159.46 (2 C-O), 162.03 and 164.46 (2 C═O, ester), 200.78 (C═O, ketone). IR (KBr) (ν_max_ /cm^-1^): 3300-3550 (OH), 1730-1740 (C═O, ketone), 1620-1640 (C═O, ester), 1400-1410 (C═C). MS, (m/z, %): 366 (5) (M+), 105 (36), 44 (100). Analysis calculated for C_21_H_18_O_6_: C 68.85, H 4.92 %. Found: C 68.80, H, 4.83%.

### Refinement

The hydrogen atom of OH group was found in difference Fourier synthesis. The other hydrogen atoms were geometrically located to the ideal positions. All hydrogen atoms were refined by using a riding model, with C—H = 0.95 and 0.98 Å and O—H = 0.94 Å and *U*_iso_(H) = 1.2 or 1.5 U_eq_(C, O).

### Crystal data

**Table:**

C_21_H_18_O_6_	*F*_000_ = 1536
*M**_r_* = 366.35	*D*_x_ = 1.372 Mg m^−^^3^
Monoclinic, *C*2/*c*	Mo *K*α radiation λ = 0.71073 Å
Hall symbol: -C 2yc	Cell parameters from 547 reflections
*a* = 22.1263 (12) Å	θ = 3–29º
*b* = 7.3012 (4) Å	µ = 0.10 mm^−^^1^
*c* = 22.5428 (12) Å	*T* = 120 (2) K
β = 103.118 (5)º	Prism, yellow
*V* = 3546.7 (3) Å^3^	0.25 × 0.20 × 0.20 mm
*Z* = 8	

### Data collection

**Table:**

Bruker SMART APEXII CCD area-detector diffractometer	4686 independent reflections
Radiation source: fine-focus sealed tube	3022 reflections with *I* > 2σ(*I*)
Monochromator: graphite	*R*_int_ = 0.034
*T* = 120(2) K	θ_max_ = 29.0º
φ and ω scans	θ_min_ = 1.9º
Absorption correction: multi-scan(APEX2; Bruker, 2005)	*h* = −29→30
*T*_min_ = 0.972, *T*_max_ = 0.981	*k* = −9→9
18827 measured reflections	*l* = −30→30

### Refinement

**Table:**

Refinement on *F*^2^	Secondary atom site location: difference Fourier map
Least-squares matrix: full	Hydrogen site location: inferred from neighbouring sites
*R*[*F*^2^ > 2σ(*F*^2^)] = 0.051	H-atom parameters constrained
*wR*(*F*^2^) = 0.132	*w* = 1/[σ^2^(*F*_o_^2^) + (0.067*P*)^2^ + 0.9*P*] where *P* = (*F*_o_^2^ + 2*F*_c_^2^)/3
*S* = 1.00	(Δ/σ)_max_ < 0.001
4686 reflections	Δρ_max_ = 0.39 e Å^−^^3^
247 parameters	Δρ_min_ = −0.24 e Å^−^^3^
Primary atom site location: structure-invariant direct methods	Extinction correction: none

### Special details

**Table:**

Geometry. All e.s.d.'s (except the e.s.d. in the dihedral angle between two l.s. planes) are estimated using the full covariance matrix. The cell e.s.d.'s are taken into account individually in the estimation of e.s.d.'s in distances, angles and torsion angles; correlations between e.s.d.'s in cell parameters are only used when they are defined by crystal symmetry. An approximate (isotropic) treatment of cell e.s.d.'s is used for estimating e.s.d.'s involving l.s. planes.
Refinement. Refinement of *F*^2^ against ALL reflections. The weighted *R*-factor *wR* and goodness of fit *S* are based on *F*^2^, conventional *R*-factors *R* are based on *F*, with *F* set to zero for negative *F*^2^. The threshold expression of *F*^2^ > σ(*F*^2^) is used only for calculating *R*-factors(gt) *etc*. and is not relevant to the choice of reflections for refinement. *R*-factors based on *F*^2^ are statistically about twice as large as those based on *F*, and *R*- factors based on ALL data will be even larger.

### Fractional atomic coordinates and isotropic or equivalent isotropic displacement parameters (Å^2^)

**Table:**

	*x*	*y*	*z*	*U*_iso_*/*U*_eq_	
O1	0.27083 (5)	0.49509 (14)	−0.00338 (4)	0.0249 (2)	
O2	0.31516 (5)	0.40901 (17)	−0.07737 (5)	0.0333 (3)	
O3	0.37885 (4)	0.81605 (14)	0.17284 (5)	0.0250 (2)	
H3O	0.3736	0.8608	0.2104	0.030*	
O4	0.47858 (5)	0.59637 (15)	0.13012 (5)	0.0293 (3)	
O5	0.45958 (5)	0.87074 (14)	0.08203 (5)	0.0245 (2)	
O6	0.32210 (5)	0.86656 (15)	0.25741 (5)	0.0269 (3)	
C1	0.32191 (7)	0.4839 (2)	−0.02871 (7)	0.0261 (3)	
C2	0.37934 (7)	0.5618 (2)	0.00649 (7)	0.0263 (3)	
H2A	0.4157	0.5540	−0.0091	0.032*	
C3	0.38259 (7)	0.6445 (2)	0.06039 (7)	0.0222 (3)	
C4	0.32758 (6)	0.66005 (19)	0.08451 (6)	0.0203 (3)	
C5	0.32623 (6)	0.74176 (19)	0.14099 (6)	0.0202 (3)	
C6	0.27130 (7)	0.74050 (19)	0.16279 (6)	0.0200 (3)	
C7	0.21878 (7)	0.6537 (2)	0.12773 (7)	0.0230 (3)	
H7A	0.1820	0.6489	0.1428	0.028*	
C8	0.21862 (7)	0.5753 (2)	0.07237 (7)	0.0230 (3)	
H8A	0.1822	0.5194	0.0489	0.028*	
C9	0.27314 (7)	0.57971 (19)	0.05135 (6)	0.0212 (3)	
C10	0.44592 (7)	0.7019 (2)	0.09627 (6)	0.0225 (3)	
C11	0.52015 (7)	0.9548 (2)	0.11350 (7)	0.0266 (3)	
C12	0.51638 (9)	1.1439 (2)	0.08531 (8)	0.0396 (4)	
H12A	0.5134	1.1328	0.0414	0.059*	
H12B	0.5537	1.2136	0.1039	0.059*	
H12C	0.4796	1.2075	0.0923	0.059*	
C13	0.52297 (8)	0.9669 (2)	0.18124 (7)	0.0300 (4)	
H13A	0.5250	0.8432	0.1985	0.045*	
H13B	0.4858	1.0292	0.1877	0.045*	
H13C	0.5600	1.0360	0.2013	0.045*	
C14	0.57332 (7)	0.8450 (3)	0.09914 (8)	0.0337 (4)	
H14A	0.5722	0.7199	0.1146	0.051*	
H14B	0.6128	0.9028	0.1186	0.051*	
H14C	0.5692	0.8415	0.0549	0.051*	
C15	0.27236 (7)	0.81611 (19)	0.22348 (7)	0.0215 (3)	
C16	0.21450 (7)	0.82855 (19)	0.24640 (6)	0.0218 (3)	
C17	0.15998 (7)	0.9054 (2)	0.21202 (7)	0.0241 (3)	
H17A	0.1586	0.9490	0.1720	0.029*	
C18	0.10785 (7)	0.9184 (2)	0.23601 (7)	0.0287 (4)	
H18A	0.0711	0.9734	0.2129	0.034*	
C19	0.10947 (8)	0.8505 (2)	0.29417 (8)	0.0307 (4)	
H19A	0.0734	0.8566	0.3103	0.037*	
C20	0.16345 (8)	0.7745 (2)	0.32831 (7)	0.0304 (4)	
H20A	0.1642	0.7276	0.3678	0.036*	
C21	0.21615 (7)	0.7663 (2)	0.30550 (7)	0.0258 (3)	
H21A	0.2536	0.7184	0.3299	0.031*	

### Atomic displacement parameters (Å^2^)

**Table:**

	*U*^11^	*U*^22^	*U*^33^	*U*^12^	*U*^13^	*U*^23^
O1	0.0239 (6)	0.0288 (6)	0.0212 (5)	−0.0018 (4)	0.0034 (4)	−0.0046 (4)
O2	0.0328 (6)	0.0417 (7)	0.0253 (6)	−0.0022 (5)	0.0067 (5)	−0.0105 (5)
O3	0.0198 (5)	0.0303 (6)	0.0236 (5)	−0.0039 (4)	0.0024 (4)	−0.0052 (4)
O4	0.0232 (6)	0.0287 (6)	0.0339 (6)	0.0006 (5)	0.0018 (5)	0.0046 (5)
O5	0.0233 (5)	0.0248 (5)	0.0244 (5)	−0.0030 (4)	0.0033 (4)	0.0003 (4)
O6	0.0237 (6)	0.0297 (6)	0.0251 (6)	−0.0004 (5)	0.0007 (4)	−0.0031 (5)
C1	0.0261 (8)	0.0265 (8)	0.0253 (8)	0.0011 (6)	0.0048 (6)	0.0002 (6)
C2	0.0225 (8)	0.0318 (8)	0.0242 (8)	0.0006 (6)	0.0048 (6)	−0.0014 (6)
C3	0.0214 (7)	0.0207 (7)	0.0236 (7)	−0.0003 (6)	0.0030 (6)	0.0032 (6)
C4	0.0196 (7)	0.0204 (7)	0.0203 (7)	−0.0003 (6)	0.0036 (6)	0.0007 (6)
C5	0.0198 (7)	0.0169 (7)	0.0226 (7)	−0.0001 (5)	0.0018 (6)	0.0008 (5)
C6	0.0203 (7)	0.0180 (7)	0.0213 (7)	0.0004 (5)	0.0040 (6)	0.0004 (5)
C7	0.0193 (7)	0.0244 (8)	0.0256 (8)	0.0005 (6)	0.0057 (6)	0.0003 (6)
C8	0.0198 (7)	0.0247 (8)	0.0229 (7)	−0.0042 (6)	0.0014 (6)	−0.0034 (6)
C9	0.0240 (8)	0.0196 (7)	0.0192 (7)	0.0012 (6)	0.0034 (6)	−0.0004 (5)
C10	0.0221 (7)	0.0266 (8)	0.0202 (7)	−0.0008 (6)	0.0079 (6)	−0.0011 (6)
C11	0.0239 (8)	0.0295 (8)	0.0251 (8)	−0.0072 (6)	0.0031 (6)	−0.0032 (6)
C12	0.0504 (11)	0.0321 (9)	0.0331 (9)	−0.0124 (8)	0.0027 (8)	0.0023 (7)
C13	0.0311 (9)	0.0340 (9)	0.0242 (8)	−0.0032 (7)	0.0051 (7)	−0.0042 (7)
C14	0.0233 (8)	0.0466 (10)	0.0323 (9)	−0.0071 (7)	0.0086 (7)	−0.0067 (8)
C15	0.0233 (7)	0.0170 (7)	0.0230 (7)	0.0013 (6)	0.0029 (6)	0.0013 (6)
C16	0.0247 (8)	0.0182 (7)	0.0224 (7)	−0.0002 (6)	0.0051 (6)	−0.0024 (6)
C17	0.0257 (8)	0.0235 (8)	0.0227 (7)	0.0003 (6)	0.0046 (6)	−0.0026 (6)
C18	0.0245 (8)	0.0267 (8)	0.0338 (9)	0.0009 (6)	0.0045 (7)	−0.0075 (7)
C19	0.0322 (9)	0.0272 (8)	0.0373 (9)	−0.0055 (7)	0.0174 (7)	−0.0096 (7)
C20	0.0456 (10)	0.0243 (8)	0.0244 (8)	−0.0047 (7)	0.0143 (7)	−0.0029 (6)
C21	0.0321 (8)	0.0209 (7)	0.0232 (7)	0.0003 (6)	0.0036 (6)	−0.0019 (6)

### Geometric parameters (Å, °)

**Table:**

O1—C9	1.3703 (17)	C11—C13	1.517 (2)
O1—C1	1.3792 (18)	C11—C14	1.518 (2)
O2—C1	1.2043 (18)	C12—H12A	0.9800
O3—C5	1.3363 (17)	C12—H12B	0.9800
O3—H3O	0.9388	C12—H12C	0.9800
O4—C10	1.2032 (17)	C13—H13A	0.9800
O5—C10	1.3259 (18)	C13—H13B	0.9800
O5—C11	1.4980 (17)	C13—H13C	0.9800
O6—C15	1.2451 (17)	C14—H14A	0.9800
C1—C2	1.453 (2)	C14—H14B	0.9800
C2—C3	1.344 (2)	C14—H14C	0.9800
C2—H2A	0.9500	C15—C16	1.488 (2)
C3—C4	1.446 (2)	C16—C17	1.395 (2)
C3—C10	1.510 (2)	C16—C21	1.400 (2)
C4—C9	1.395 (2)	C17—C18	1.384 (2)
C4—C5	1.412 (2)	C17—H17A	0.9500
C5—C6	1.411 (2)	C18—C19	1.395 (2)
C6—C7	1.400 (2)	C18—H18A	0.9500
C6—C15	1.471 (2)	C19—C20	1.382 (2)
C7—C8	1.372 (2)	C19—H19A	0.9500
C7—H7A	0.9500	C20—C21	1.378 (2)
C8—C9	1.393 (2)	C20—H20A	0.9500
C8—H8A	0.9500	C21—H21A	0.9500
C11—C12	1.514 (2)		
			
C9—O1—C1	122.18 (11)	C11—C12—H12A	109.5
C5—O3—H3O	110.8	C11—C12—H12B	109.5
C10—O5—C11	119.65 (11)	H12A—C12—H12B	109.5
O2—C1—O1	117.38 (14)	C11—C12—H12C	109.5
O2—C1—C2	125.99 (15)	H12A—C12—H12C	109.5
O1—C1—C2	116.63 (13)	H12B—C12—H12C	109.5
C3—C2—C1	122.02 (14)	C11—C13—H13A	109.5
C3—C2—H2A	119.0	C11—C13—H13B	109.5
C1—C2—H2A	119.0	H13A—C13—H13B	109.5
C2—C3—C4	119.89 (13)	C11—C13—H13C	109.5
C2—C3—C10	117.50 (13)	H13A—C13—H13C	109.5
C4—C3—C10	122.35 (12)	H13B—C13—H13C	109.5
C9—C4—C5	117.94 (13)	C11—C14—H14A	109.5
C9—C4—C3	117.63 (13)	C11—C14—H14B	109.5
C5—C4—C3	124.33 (13)	H14A—C14—H14B	109.5
O3—C5—C6	122.00 (13)	C11—C14—H14C	109.5
O3—C5—C4	117.50 (13)	H14A—C14—H14C	109.5
C6—C5—C4	120.49 (13)	H14B—C14—H14C	109.5
C7—C6—C5	118.47 (13)	O6—C15—C6	120.52 (13)
C7—C6—C15	122.08 (13)	O6—C15—C16	118.61 (13)
C5—C6—C15	119.25 (13)	C6—C15—C16	120.84 (13)
C8—C7—C6	122.19 (14)	C17—C16—C21	119.46 (14)
C8—C7—H7A	118.9	C17—C16—C15	122.35 (13)
C6—C7—H7A	118.9	C21—C16—C15	118.15 (13)
C7—C8—C9	118.44 (13)	C18—C17—C16	120.18 (14)
C7—C8—H8A	120.8	C18—C17—H17A	119.9
C9—C8—H8A	120.8	C16—C17—H17A	119.9
O1—C9—C8	115.97 (13)	C17—C18—C19	119.81 (15)
O1—C9—C4	121.56 (13)	C17—C18—H18A	120.1
C8—C9—C4	122.44 (13)	C19—C18—H18A	120.1
O4—C10—O5	127.73 (14)	C20—C19—C18	120.06 (15)
O4—C10—C3	120.85 (13)	C20—C19—H19A	120.0
O5—C10—C3	111.30 (12)	C18—C19—H19A	120.0
O5—C11—C12	102.51 (12)	C21—C20—C19	120.49 (14)
O5—C11—C13	109.18 (12)	C21—C20—H20A	119.8
C12—C11—C13	110.81 (13)	C19—C20—H20A	119.8
O5—C11—C14	109.65 (12)	C20—C21—C16	119.93 (15)
C12—C11—C14	111.31 (14)	C20—C21—H21A	120.0
C13—C11—C14	112.87 (13)	C16—C21—H21A	120.0
			
C9—O1—C1—O2	−179.04 (13)	C5—C4—C9—C8	−1.0 (2)
C9—O1—C1—C2	1.8 (2)	C3—C4—C9—C8	175.46 (13)
O2—C1—C2—C3	179.48 (16)	C11—O5—C10—O4	−5.1 (2)
O1—C1—C2—C3	−1.5 (2)	C11—O5—C10—C3	178.97 (11)
C1—C2—C3—C4	−0.8 (2)	C2—C3—C10—O4	−86.24 (18)
C1—C2—C3—C10	173.41 (14)	C4—C3—C10—O4	87.86 (18)
C2—C3—C4—C9	2.8 (2)	C2—C3—C10—O5	89.97 (16)
C10—C3—C4—C9	−171.14 (13)	C4—C3—C10—O5	−95.93 (16)
C2—C3—C4—C5	179.01 (14)	C10—O5—C11—C12	−178.97 (13)
C10—C3—C4—C5	5.0 (2)	C10—O5—C11—C13	−61.43 (17)
C9—C4—C5—O3	179.06 (13)	C10—O5—C11—C14	62.70 (17)
C3—C4—C5—O3	2.9 (2)	C7—C6—C15—O6	168.75 (14)
C9—C4—C5—C6	0.3 (2)	C5—C6—C15—O6	−6.0 (2)
C3—C4—C5—C6	−175.90 (13)	C7—C6—C15—C16	−9.3 (2)
O3—C5—C6—C7	−177.56 (13)	C5—C6—C15—C16	175.93 (13)
C4—C5—C6—C7	1.2 (2)	O6—C15—C16—C17	132.22 (15)
O3—C5—C6—C15	−2.6 (2)	C6—C15—C16—C17	−49.7 (2)
C4—C5—C6—C15	176.11 (13)	O6—C15—C16—C21	−45.45 (19)
C5—C6—C7—C8	−2.0 (2)	C6—C15—C16—C21	132.65 (15)
C15—C6—C7—C8	−176.82 (14)	C21—C16—C17—C18	−0.6 (2)
C6—C7—C8—C9	1.4 (2)	C15—C16—C17—C18	−178.23 (13)
C1—O1—C9—C8	−177.93 (13)	C16—C17—C18—C19	−1.5 (2)
C1—O1—C9—C4	0.2 (2)	C17—C18—C19—C20	1.5 (2)
C7—C8—C9—O1	178.28 (13)	C18—C19—C20—C21	0.5 (2)
C7—C8—C9—C4	0.2 (2)	C19—C20—C21—C16	−2.6 (2)
C5—C4—C9—O1	−178.97 (13)	C17—C16—C21—C20	2.6 (2)
C3—C4—C9—O1	−2.5 (2)	C15—C16—C21—C20	−179.65 (13)

### Hydrogen-bond geometry (Å, °)

**Table:**

*D*—H···*A*	*D*—H	H···*A*	*D*···*A*	*D*—H···*A*
O3—H3O···O6	0.94	1.72	2.5365 (15)	143
